# In this month’s Bulletin

**DOI:** 10.2471/BLT.17.001117

**Published:** 2017-11-01

This month’s issue has a special focus on healthy ageing, as introduced by John R Beard et al. (730) in the first editorial. Dulce M Cruz-Oliver et al. (731) expand on this theme with a discussion of end-of-life care in low and middle-income countries. 

Vijay Shankar Balakrishnan and Fiona Fleck (734-735) report on efforts to improve training and support for caregivers of people with dementia in Ghana, India and Lebanon. Andréia Azevedo Soares (736–737) interviews Yuji Kuroiwa about technological and political approaches to the ageing population in Japan. 

## Bangladesh, China, India, Singapore

### Holding children responsible for their eldery parents. 

Ray Serrano et al. (788–790) examine filial support laws for fairness and reciprocity.

## Bangladesh, Haiti, Kenya, Malawi, Namibia, Nepal, Rwanda, Senegal, Uganda, United Republic of Tanzania 

### Can the clinic provide care?

Hannah H Leslie et al. (738–749) assess service readiness of health facilities.

## Costa Rica, Thailand

### Meeting the needs of elderly people

Peter Lloyd-Sherlock et al. (774–778) consider volunteer provision of long-term care.

## Sweden

### Mitigating the harms of antibiotics

Sigvard Mölstad et al. (764–773) collate lessons learnt from two decades of preventing antimicrobial resistance. 

## Tanzania

### Dying from a vaccine-preventable disease

Riaz Aziz et al. (779–783) report tetanus deaths in men. 

## Global

### Benefit without coercion

Belinda Bennett et al. (750–755) explore the implications of technology used to assist elderly people.

### Designing care for older people

Islene Araujo de Carvalho et al. (756–763) describe WHO’s approach to transforming health services.

### Tracking accountability 

Frank Pega et al. (784–787) explain the need to monitor action on the social determinants of health.

### When does old age start?

Sridhar Venkatapuram et al. (791–792) question assumptions about chronological and functional ageing.

